# Corticofugal Modulation of Initial Neural Processing of Sound Information from the Ipsilateral Ear in the Mouse

**DOI:** 10.1371/journal.pone.0014038

**Published:** 2010-11-16

**Authors:** Xiuping Liu, Yuchu Yan, Yalong Wang, Jun Yan

**Affiliations:** 1 Health Science Centre, Hebei University, Baoding, People's Republic of China; 2 Department of Physiology and Pharmacology, Hotchkiss Brain Institute, Faculty of Medicine, University of Calgary, Alberta, Canada; University of Auckland, New Zealand

## Abstract

**Background:**

Cortical neurons implement a high frequency-specific modulation of subcortical nuclei that includes the cochlear nucleus. Anatomical studies show that corticofugal fibers terminating in the auditory thalamus and midbrain are mostly ipsilateral. Differently, corticofugal fibers terminating in the cochlear nucleus are bilateral, which fits to the needs of binaural hearing that improves hearing quality. This leads to our hypothesis that corticofugal modulation of initial neural processing of sound information from the contralateral and ipsilateral ears could be equivalent or coordinated at the first sound processing level.

**Methodology/Principal Findings:**

With the focal electrical stimulation of the auditory cortex and single unit recording, this study examined corticofugal modulation of the ipsilateral cochlear nucleus. The same methods and procedures as described in our previous study of corticofugal modulation of contralateral cochlear nucleus were employed simply for comparison. We found that focal electrical stimulation of cortical neurons induced substantial changes in the response magnitude, response latency and receptive field of ipsilateral cochlear nucleus neurons. Cortical stimulation facilitated auditory response and shortened the response latency of physiologically matched neurons whereas it inhibited auditory response and lengthened the response latency of unmatched neurons. Finally, cortical stimulation shifted the best frequencies of cochlear neurons towards those of stimulated cortical neurons.

**Conclusion:**

Our data suggest that cortical neurons enable a high frequency-specific remodelling of sound information processing in the ipsilateral cochlear nucleus in the same manner as that in the contralateral cochlear nucleus.

## Introduction

Neurons in layers V and VI of the auditory cortex send numerous descending fibers to the auditory thalamus, midbrain and cochlear nucleus (CN) [1]–[5]. Neurophysiological studies have demonstrated that cortical neurons alter the auditory responses and receptive fields of subcortical neurons via corticofugal projections in a frequency-specific manner [6]–[10]. Briefly, focal activation of cortical neurons facilitates physiologically matched subcortical neurons but inhibits unmatched neurons. This activation also shifts the receptive fields of subcortical neurons towards those of activated cortical neurons [11]–[18]. Therefore, sound information favored by cortical neurons is preferentially selected in the subcortical nuclei prior to higher-level cortical processing. While previous corticofugal studies provided insight into these mechanisms, a potential shortcoming is their singular focus on the subcortical nuclei along the ascending pathway arising from the contralateral ear.

Anatomical studies show that corticofugal fibers terminating in the auditory thalamus are entirely ipsilateral while those terminating in the auditory midbrain are predominantly but not exclusively ipsilateral [1], [19]. In other words, corticofugal neurons mainly innervate the auditory thalamus and midbrain in one ascending pathway. In contrast, corticofugal innervations of the CN are bilateral. One-third of the corticofugal fibers terminate in the contralateral CN and approximately two-thirds terminate in the ipsilateral CN [20]. These specialized features of the corticofugal projections suggest that cortical neurons in one hemisphere may modulate the neural processing of acoustic signals arising from both ears. An understanding of the corticofugal projections to the CN is of particular interest in terms of their role in top-down selection (filtering) and binaural hearing in auditory cognition and/or comprehension [21]–[28].

Our recent study confirmed that focal cortical activation selectively enhances neural processing of specific auditory information and attenuates others in the contralateral CN based on sound frequencies encoded in the auditory cortex [18]. Still, it remains unclear as to how cortical neurons modulate the ipsilateral CN. This study attempts to clarify this issue in order to understand the coordination of corticofugal modulation of the bilateral auditory ascending pathways. Our data show that focal electrical stimulation (ES) of the primary auditory cortex (AI) resulted in highly frequency-specific changes in auditory responses and receptive fields of ipsilateral CN neurons. The results are largely identical to our previous examinations of contralateral CN neurons [18].

## Results

For comparison, the methods and procedures used in this study were the same as those used in our previous study of corticofugal modulation of contralateral CN [18]. Briefly, focal electrical stimulation (ES) of the AI (ES_AI_) of the C57 mouse was used for cortical activation. Recording electrode was placed in the anteroventral CN. Our data were collected using two different procedures. The first group of results represents the responses of CN neurons to a series of tone bursts at different frequencies and at the minimum threshold of a given neuron. Responses of the CN neurons were continuously measured before, during and after ES_AI_. The second group represents the frequency response curves (receptive fields) of CN neurons prior to and following ES_AI_. In total, the effects of ES_AI_ on 56 CN neurons were examined.

**Figure 1:**
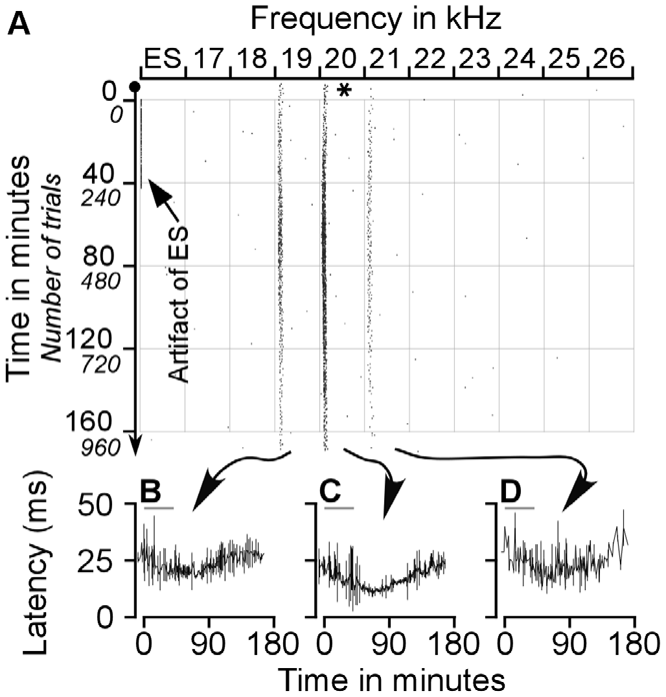
Effect of ES_AI_ on the auditory responses of a BF-matched CN neuron sampled with protocol 1. **A**. Raster plot of typical changes in auditory responses of a CN neuron with the same BF as that of AI neuron before, during, and after ES_AI_. The responses to 19–20 kHz tone markedly increased following ES_AI_. **B–D**. Changes in the latencies of this neuron in response to 19 kHz, 20 kHz and 21 kHz tones following ES_AI_. The response latencies were reduced by ES_AI_. The asterisk represents the BF of the stimulated AI neurons. The grey lines in B–D represent ES_AI_.

**Figure 2 pone-0014038-g002:**
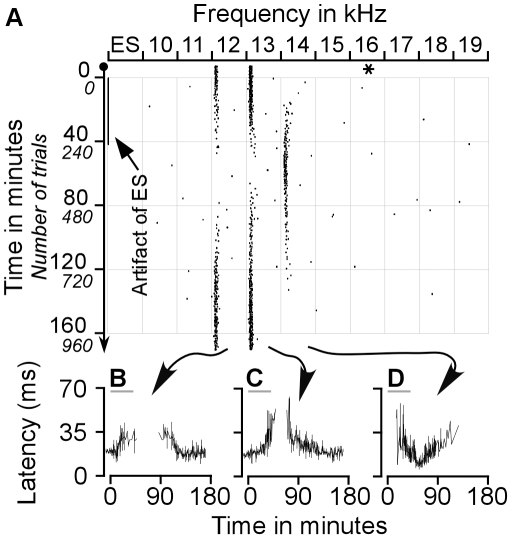
Effect of ES_AI_ on the auditory responses of a BF-unmatched CN neuron sampled with protocol 1. **A**. Raster plotting of a typical change in auditory responses of a CN neuron that had a BF different from that of AI neurons before, during, and after ES_AI_. The responses to 12–13 kHz decreased and then ceased whereas the responses to 14 kHz appeared and gradually increased following electrical stimulation of AI neuron tuned 16 kHz. **B–D**. Changes in the latencies of this neuron in response to 12 kHz, 13 kHz and 14 kHz tones following ES_AI_. The response latencies to 12–13 kHz tones gradually increased before the cessation of auditory responses and recovered with extremely long latencies. The changes in response latencies were similar to that of BF-matched neurons depicted in Fig. 1. The asterisk represents the BF of the stimulated AI neurons. The grey lines in B–D represent ES_AI_.

Two examples of our first group of results are presented in Figures 1A and 2A. Figure 1A demonstrates ES_AI_-evoked changes in auditory responses of a CN neuron with a BF identical to that of the stimulated AI neurons, i.e., a BF-matched CN neuron. In contrast, Figure 2A shows a CN neuron with a BF different from that of the stimulated AI neurons, i.e., a BF-unmatched CN neuron.

The neuron in Figure 1 shows responses to tone stimuli ranging from 19 to 21 kHz. Its maximum response was to 20-kHz tone, representing the BF of this neuron. When AI neurons tuned to 20 kHz were electrically stimulated, responses to 19–21 kHz tones gradually increased. Forty-two minutes after the onset of the ES_AI_, the responses of the CN neuron to these three frequencies continued to increase. Its response magnitude to a 20-kHz tone reached a maximum at 83 minutes after the onset or at 41 minutes after the cessation of the ES_AI_. At this juncture, the responses elicited by the three frequencies began to decline. The responses reverted to nearly the control level approximately 160 min. after the onset of the ES_AI_. Throughout the duration of data collection, the response magnitude evoked by 20-kHz tone remained greater than at any other frequency. This demonstrates that the BF of this neuron remained constant.

In addition to the increase in response magnitude, Figure 1A shows changes in the response latency. We measured the averaged response latency of all spikes within every one-minute time frame; the results are presented in the bottom panel of Figure 1(B–D ). The response latency preceding the ES_AI_ was 25.83±10.65 ms with 19 kHz, 22.75±7.61 ms with 20 kHz and 28.50±8.98 with 21 kHz. ES_AI_ gradually decreased the response latencies of this CN neuron in all three frequencies. The shortest response latency of this neuron to 19 kHz was observed at 75 minutes (17.16±0.58 ms), to 20 kHz at 83 minutes (11.18±0.6 ms) and to 21 kHz at 64 minutes (12.50±0.71 ms) after the onset of ES_AI_. These response latencies gradually lengthened during successive time intervals (Fig. 1, B–D).

The example shown in Figure 2 exhibited more remarkable changes following the ES_AI_. This neuron originally showed responses to 12-kHz and 13-kHz tones but did not respond to tones of other frequencies. The largest response was induced by the 13-kHz tone, representing the BF of the neuron. During and after the electrical stimulation of cortical neurons tuned to 16 kHz, the auditory responses of the CN neuron exhibited drastic changes in response to 12–14 kHz tones (Fig. 2A). During the ES_AI_, the responses of this neuron to 12-kHz and 13-kHz tones gradually declined and finally ceased. The responses to 12-kHz and 13-kHz respectively ceased at 44 minutes and 49 minutes after the onset of the ES_AI_ or at 2 minutes and 7 minutes after the ES_AI_ stopped. Its responses to the 13-kHz tone started resuming at 28 minutes, while those to 12-kHz started at 41 minutes after ES_AI_ cessation. In contrast, responses to the 14-kHz tone were initially observed at 16 minutes following the onset of the ES_AI_. The response reached the maximum at 56 minutes after the onset or 14 minutes after the cessation of the ES_AI_. Over time, this neuron gradually reduced its firing rate in response to the 14-kHz tone and became silent at 132 minutes after the onset or 90 minutes after the cessation of the ES_AI_. Within the time interval of 30–86 minutes following the ES_AI_, the neurons reacted most vigorously to the 14-kHz tone; the BF of this neuron shifted toward 16 kHz, the BF of stimulated cortical neurons. By 160 minutes following the ES_AI_ or 118 minutes after termination of the stimulation, neuronal responses to 12–14 kHz tones had reverted to pre-stimulation levels.

Changes in response latencies accompanied the above changes in response magnitudes. Initial response latencies to 12-kHz and 13-kHz tones were 21.33±7.56 ms and 17.67±7.51 ms respectively. Prior to the termination of ES_AI_ delivery, the response latencies increased sharply, and then resumed with longer latencies. Gradually, latencies shortened to 20.50±3.22 ms and 19.67±4.62 ms at 160 minutes following the ES_AI_ or 118 minutes after withdrawal of the ES_AI_ (Fig. 2, B and C). The response latency to 14-kHz tone was initially about 50 ms. The shortest latency was 18.33±2.08 ms achieved at 56 minutes following initial ES_AI_ or 14 minutes following ES_AI_ removal. Subsequently, the response latency increased steadily, reaching a latency of 45 ms by the last spike (Fig. 2D).

In total, 16 CN neurons from 16 mice were examined using this procedure. Out of the16 CN neurons studied, 8 were BF-matched while the remaining 8 were BF-unmatched neurons. BFs of matched neurons differed from those of stimulated cortical neurons by less than 1 kHz. In contrast, BFs of unmatched neurons differed from those of the stimulated cortical neurons by greater than 1 kHz.

Out of 8 BF-matched neurons, 6 neurons showed similar changes to that displayed in Figure 1, 1 neuron demonstrated a decrease in response magnitude, and another shifted its BF by 1 kHz. Out of 8 BF-unmatched neurons, only 2 neurons exhibited the response cessation to the control BFs. Both neurons showed extremely long response latencies immediately before and after the cessation period (Fig. 2). The remaining 6 neurons displayed a remarkable decrease in response to the control BFs. All BF-unmatched neurons exhibited BF shifts toward that of the stimulated cortical neurons. BF shifts stemmed from the decrease in responses to control BF and an increase in responses to the shifted BF. The response magnitude and BF changes were analyzed at a later time, a procedure previously employed in other studies [15], [16], [18]. We limited our analysis of changes in the latencies to the control BF (pre-stimulus BF) of a given neuron following the ES_AI_.

**Figure 3:**
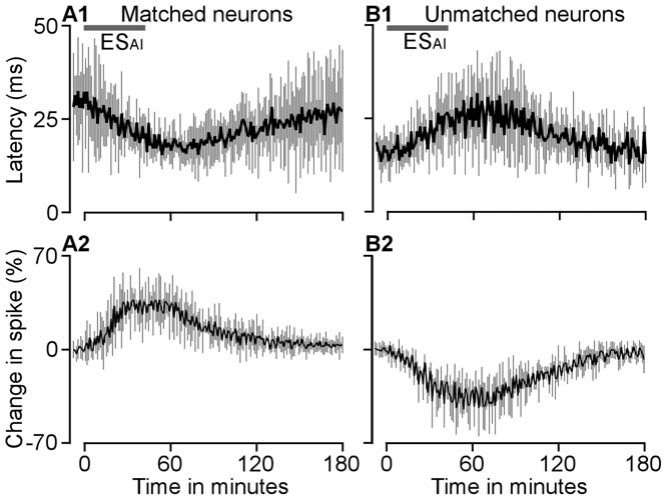
Changes in response latencies and magnitudes of BF-matched (A1 and A2) and BF-unmatched (B1 and B2) CN neurons to the time interval from onset of the ES_AI_. Data presented were sampled with protocol 1. The grey bars represent ES_AI_.

On average, the latency of BF-matched CN neurons rapidly decreased during the ES_AI_. The latency began a gradual recovery once the shortest latency was achieved (Fig. 3A1). In contrast, the latency of BF-unmatched CN neurons increased during the ES_AI_, then steadily returned to the pre-stimulus level (Fig. 3B1). Similar time courses were also seen in the percentage change in response magnitude. Cortical stimulation gradually increased the spike number of matched neurons (Fig. 3A2) whereas it decreased that of unmatched neurons (Fig. 3B2). The changes in spike number gradually returned to the pre-stimulus level after reaching a peak.

**Figure 4:**
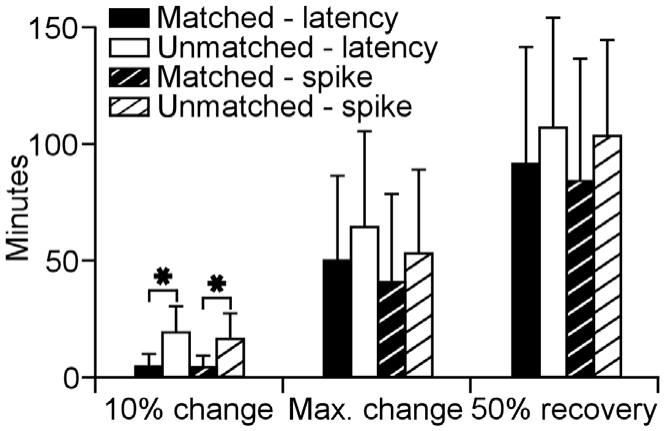
Time required for 10% change, maximum changes, and 50% recovery in response latencies and magnitudes of the BF-matched and BF-unmatched neurons after ES_AI_. Data presented were sampled with protocol 1. *: *p*<0.001, compared between matched and unmatched neurons.

The 10% change in response latency of BF-matched neurons occurred significantly earlier than that of unmatched neurons (3.75±5.86 minutes vs. 18.62±11.48 minutes, *p*<0.05). The time that latency change reached a peak was 49.12±36.41 minutes for matched neurons, which was shorter than that for unmatched neurons (63.37±41.22 minutes) but no statistical difference was found (*p*>0.05). On average, the latency changes recovered by 50% at post-stimulus 90.32±50.87 minutes for matched neurons and 106.47±47.10 for unmatched neurons (*p*>0.05). Similarly, the time correlated with a 10% change in the auditory response of BF-matched neurons was 3.35±6.24 minutes after the ES_AI_ which was significantly shorter than that of BF-unmatched neurons (15.64±11.48 minutes, *p*<0.001). The change in response magnitude of BF-matched neurons peaked (the shortest latency) at 40.64±39.56 minutes and declined by 50% at 83.45±52.74 minutes after the ES_AI_. In comparison, changes in response magnitude of BF-unmatched neurons reached the maximum at 52.88±36.35 minutes and declined by 50% at 102.37±41.68 minutes after the ES_AI_. The differences between the peak changes and 50% recovery rates of matched and unmatched CN neurons were insignificant (*p*>0.05, Fig. 4).

When comparing the time courses of the changes in response latency and magnitude, our data indicated that the averaging times of 10% change, maximum change and 50% recovery in matched neurons were shorter than those in unmatched neurons. Statistical analysis however, suggested that there were no differences between them (*p*>0.05).

**Figure 5:**
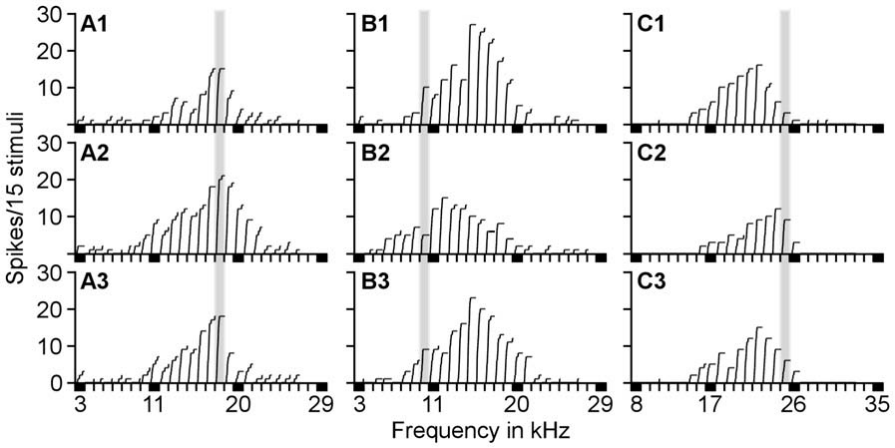
Three examples of the effects of ES_AI_ on frequency tunings of CN neurons sampled with protocol 2. ES_AI_ did not change the BFs but augmented the auditory responses of BF-matched CN neurons (**A**) whereas ES_AI_ shifted the BFs of BF-unmatched CN neurons toward the cortical BFs (**B, C**). The grey bars represent the BFs of the stimulated cortical neurons.

We selected three 3 CN neurons to exemplify the impact of the ES_AI_ on CN frequency tunings. The first example showed a neuron originally tuned to 18 kHz (Fig. 5A1). Electrical stimulation of 18-kHz-tuned AI neurons did not alter the BF of this CN neuron but increased its spike number (Fig. 5A2). The second example demonstrates a neuron originally tuned to 15 kHz (Fig. 5B1). When AI neurons tuned to 10 kHz were electrically stimulated, its BF shifted to 11 kHz, a downward shift of 4 kHz toward the BF of stimulated AI neurons. The response magnitude of this CN neuron to the original BF (15 kHz) was significantly declined (Fig. 5B2). The third example shows a neuron originally tuned to 22 kHz (Fig. 5C1). When 25-kHz-tuned AI neurons were electrically stimulated, the BF of this CN neuron shifted to 24 kHz, an upward shift of 2 kHz toward the BF of the stimulated AI neurons. Its response magnitude to the original BF was also declined (Fig. 5C2).

**Figure 6:**
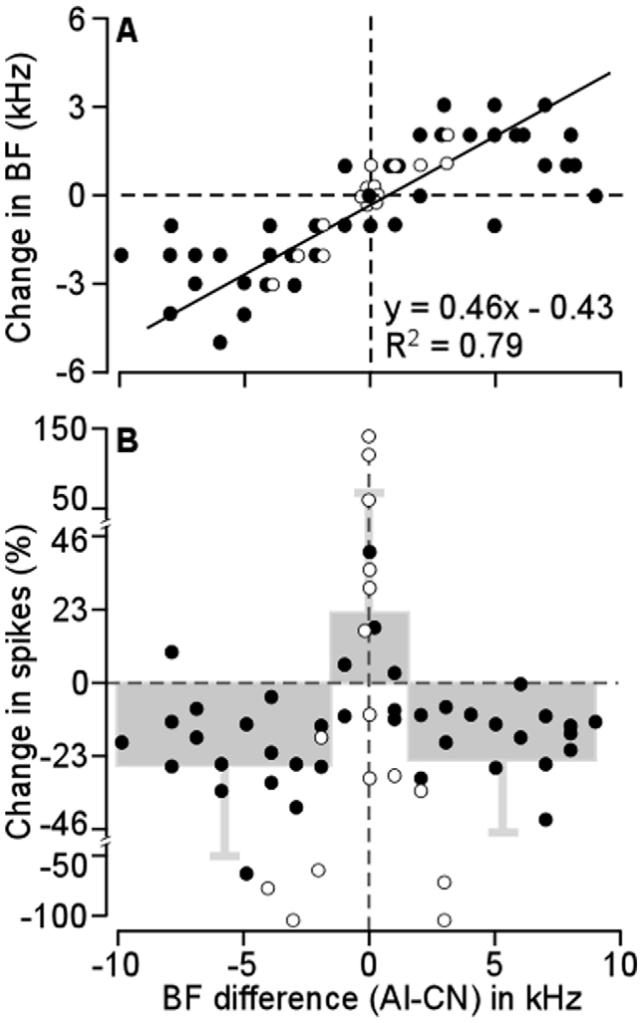
The changes in BFs (A) and response magnitudes (B) of CN neurons induced by ES_AI_ as the function of BF differences between the stimulated AI and measured CN neurons. These changes of BF-matched and BF-unmatched CN neurons were clearly different. The line in A is a regression line. Open circles represent data sampled with protocol 1 and filled circles represent data sampled with protocol 2. The grey boxes and bars in B represent the mean ± SD of percentage changes in spike number following ES_AI_.

Forty CN neurons were studied using this protocol. The frequency-dependent changes were analyzed by combining these neurons with 16 additional neurons sampled from protocol 1. The BFs of the majority of these CN neurons were altered by the ES_AI_. The BF changes were directly impacted by the BFs of the recorded CN and stimulated AI neurons. When BFs of the AI neurons were higher than those of the CN neurons, the BFs of the CN neurons increased. The opposite was observed when the BFs of the AI neurons were lower than the CN neurons. CN neurons expressed minute changes in their BFs when they were similar to those of the stimulated AI neurons. In other words, ES_AI_ shifted the BFs of the CN neurons toward those of the AI neurons. There was a linear relationship between the BF shift of CN neurons and the difference of CN BFs from AI BFs (Fig. 6A). The difference in AI and CN BFs also determined changes in the response magnitude of CN neurons following the ES_AI_. Spike numbers of CN neurons decreased when the CN BFs were different from those of AI neurons whereas they increased when the CN BFs were similar to the AI BFs (Fig. 6B). On average, the spike numbers of CN neurons declined by 26.91±24.47% when AI BFs exceeded CN BFs and by 24.93±23.89% when AI BFs were below CN BFs. Spike numbers showed an average increase of 22.22±50.31% when BFs of CN and AI neurons were similar.

**Figure 7:**
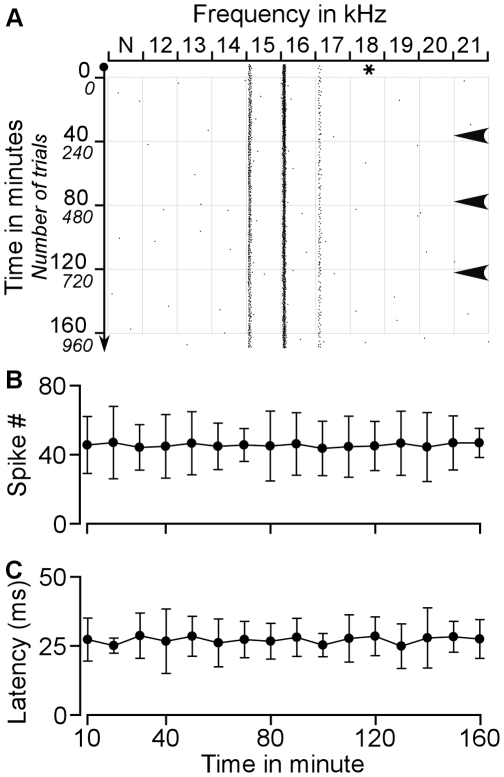
Effects of sham stimulation of the AI on the auditory responses of CN neuron. **A**. Raster plot of typical changes in auditory responses of a CN neuron when a stimulating electrode was placed in the AI but no electrical stimulation was given. **B**. Averaged spike numbers calculated within a 10-minute window during the observation period. **C**. Averaged response latencies calculated within a 10-minute window during the observation period. The asterisk represents the BF of the AI neuron where the electrode was placed. Arrowheads indicate supplementary injection of ketamine and xylazine. N: no electrical stimulation.

**Figure 8:**
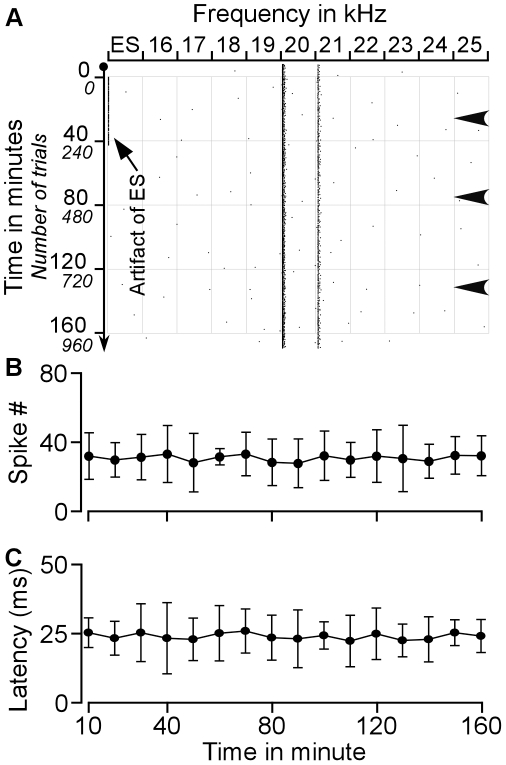
Effects of electrical stimulation of non-AI cortical area on the auditory responses of CN neuron. **A**. Raster plot of typical changes in auditory responses of a CN neuron before, during and after electrical stimulation. **B**. Averaged spike numbers calculated within a 10-minute window during the observation period. **C**. Averaged response latencies calculated within a 10-minute window during the observation period. Arrowheads indicate supplementary injection of ketamine and xylazine. ES: electrical stimulation. N: no electrical stimulation.

Two control experiments were preformed to clarify if the changes in auditory response, latency and BF of CN neurons described above did result from the ES_AI_. One was sham stimulation of the AI; the stimulating electrode was placed in the AI but no electrical stimulation was given. Five CN neurons sampled from 5 mice were observed. An example shown in Figure 7A exhibited responses to tones of 15–17 kHz and the response to 16 kHz was the strongest. The response magnitude and latency of this neuron were quite constant during the observation period. Out of 5 studied neurons, none of them showed significant fluctuation in spike number (Fig. 7B) and response latency (Fig. 7C) and the BFs of these neurons were not changed. The other control experiment employed electrical stimulation of non-AI cortical area. We placed the stimulating electrode in the cortical area dorsal to the auditory cortex. Five CN neurons from 5 mice were studied. A CN neuron shown in Figure 8A exemplifies the impact of non-AI stimulation on the auditory responses of CN neurons. This neuron was tuned to 20 kHz. Electrical stimulation of non-AI cortical area did not change the BF and had little effect on the response magnitude and latency. Out of 5 studied neurons, none of them exhibited a changes in BF and the fluctuations in response magnitude (Fig. 8B) and latency (Fig. 8C) were in significant.

Since little fluctuation in response magnitude and latency was observed, these 10 neurons were also used to examine the effects of anaesthesia on the auditory responses of CN neurons. The arrowheads in Figures 7A and 8A indicate supplementary injection of ketamine and xylazine. Rare changes in spike number and response latency were shown. Out of 37 injections (3 to 4 injections per animal during the observation period), obvious change in spike number was observed in 5 cases. We calculated the 10-minute spike number immediately before and after the supplementary injection of aesthetic drugs. On average, the spike number was 39.14±9.7 before and 37.08±11.4 after the supplementary injection (*p*>0.05) and the latency was 26.05±7.98 ms before and 27.32±7.62 ms after the supplementary injection (*p*>0.05).

## Discussion

In the natural environment, sounds from multiple sources impinge both eardrums. Listeners are able to focus on a specific sound source even though complex acoustic signals enter the brain via the auditory nerve [22], [23]. The brain must select one incoming signal and eliminate or attenuate others. Automatic sound selection is critical because it reduces information overload and allows efficient processing of relevant sound [29]. Top-down selection or filtering is the current working model for automatic sound selection in the brain and is an active process involving in intention, expectation, or experience [24]–[28]. Physiological studies in the past 15 years have revealed that systematically organized descending projections from the auditory cortex modulate subcortical sound processing in a highly selective manner [6], [7], [9], [10]. Cortical activation facilitates subcortical neurons with the same receptive fields as those of activated cortical neurons, but inhibits those with different receptive fields from those of activated cortical neurons [11]–[15], [17]. The auditory cortex also modulates the microphonic potential of the inner ear in a frequency-specific manner [30], [31]. Such specific downward modulation appears to be strengthened in a series of steps; the tuning shifts induced by cortical activation intensify as they progress from the cochlear nucleus to the auditory midbrain to the auditory thalamus [12], [15], [18], [32]. These findings suggest that the corticofugal system functions as a highly selective back-projection system and could be a neural substrate in the top-down selection pathway/process [18], [24], [26].

Sound localization is a fundamental function of hearing. Many neurons in the central auditory system respond to sound information from both ears to encode the azimuth of the sound source. These neurons are called binaural neuron and initially emerge from the superior olivery complex based on information from both ipsilateral and contralateral CNs. The response latency and magnitude information from CNs are particularly important since the azimuth of the sound source is represented by interaural time difference tuning and interaural intensity difference tuning of binaural neurons [33]–[36]. Our data, which shows the equivalence of corticofugal modulation of the response latency and magnitude in both CNs, strongly suggest that such top-down modulation does not change the azimuth coding of binaural neurons in the central auditory system but rather, enhances that of the cortex-selected sound source.

Binaural hearing also improves the quality of hearing and vocal communication in addition to sound localization. Psychoacoustic studies have long demonstrated that acoustic information from two ears improves perception and speech comprehension in a natural environment [21], [37], [38]. Clinical research also indicate that compared to unilateral cochlear implantation, bilateral cochlear implantation enhances sound localization, speech intelligibility, communication and listening comprehension of deaf patients in reverberant and noisy environments [39]. Therefore, it is entirely plausible if corticofugal modulation of neural processing of sound information from both ears can be comparable, equivalent or coordinated.

Corticofugal projections to the auditory thalamus and midbrain are mostly unilateral whereas those to the cochlear nucleus are bilateral [1], [4], [19], [20]. Our previous study revealed that cortical neurons implement frequency-specific modulation of the contralateral cochlear nucleus [18]. The auditory cortex modulates the contralateral cochlear nucleus through two specific mechanisms. One is auditory response enhancement and response latency shortening in physiologically matched neurons; the opposite stands for physiologically unmatched neurons. A second mechanism is the enhancement of neural representations of cortex-favoured sounds, evident in the shift of CN BFs towards those of activated cortical neurons. Data from this study as well as our earlier work confirm that corticofugal modulation of the ipsilateral and contralateral cochlear nuclei is very similar. The similarity is briefly explained by the following: First of all, cortical activation facilitates the auditory responses of matched neurons but inhibits those of unmatched neurons (Figs. 1–2). Second, cortical activation shortens the response latencies of matched neurons while lengthening those of unmatched neurons (Figs. 1–3). Third, the time required for cortical stimulation to evoke a change of 10% is significantly shorter for matched than that for unmatched neurons (Fig. 4). Fourth, times required for maximum change and 50% recovery do not differ between matched and unmatched neurons (Fig. 4). Fifth, cortical activation systematically shifts neuronal BFs toward the BFs of cortical neurons (Fig. 5). The degrees of BF shifts are equivalent and so are the slopes of regression lines which are 0.41 and 0.46 for contralateral [18] and ipsilateral (Fig. 6A) cochlear nuclei, respectively. Last, the response magnitudes are systematically related to the BF differences between cortical and CN neurons (Fig. 6B). Our work suggests that corticofugal modulations of both ascending pathways are comparable or coordinated at the first auditory processing center. Furthermore, we propose that the corticofugal system could be the neural substrate that comprises or satisfies requirements of automatic sound selection and binaural hearing in the natural acoustic environment [18].

Our findings further raise a number of interesting issues that require clarification. First, our current understanding of the neural mechanism underlying corticofugal modulation remains poor. In general, the corticofugal fibers should be excitatory since global inactivation of the primary auditory cortex leads to nonspecific deduction of the auditory responses of midbrain neurons [40], [41]. Studies in the visual and somatosensory system indicate that the neurotransmitter of corticofugal fibers is glutamine and that the postsynaptic receptor is possibly the metabotropic glutamate receptor or the N-methyl-D-aspartate receptor [42]–[45]. Likely, glutamine is the neurotransmitter for the corticofugal projection to the cochlear nucleus and corticofugal modulation could be mediated by glutamate receptors. This helps to explain the corticofugal facilitation of matched CN neurons such as the increase in response magnitude and decrease in response latency. The corticofugal effects on unmatched neurons remain unclear. Our data showed that the corticofugal effects on matched neurons occurred earlier than that on unmatched neurons. One possibility is that the change in the response magnitude and latency of unmatched neurons is a consequence of the facilitation of matched neurons through local neural circuitry. Second, recent studies indicate that focal cortical stimulation results in highly specific remodelling of the neuronal receptive fields of the contralateral auditory cortex, presumably via the corpus callosum [46], [47]. This raises the contention that the highly specific neuronal remodelling of the ipsilateral CN evoked by cortical activation may also be mediated by descending projections from the contralateral auditory cortex to the CN. Third, in addition to the direct projections from the cortex to the CN, cortical neurons also send projections to other subcortical nuclei such as the inferior colliculus of the midbrain and superior olivary complex of the low brainstem, which in turn send descending projections to the CN [48]–[51]. Therefore, both direct and indirect descending projections could be involved in the corticofugal modulation of CN neurons. The function of descending projections from subcortical nuclei is rarely reported. One study involving the moustached bat demonstrates that electrical stimulation of the central nucleus of the inferior colliculus sharpens and shifts the frequency tuning of cochlear hair cells [52]. This finding, reported in a book chapter by Suga and his colleagues, suggests that colliculofugal modulation is similar to corticofugal modulation. Additional investigations are required to advance our understanding of subcortical descending projections and their function. Fourth, corticofugal fibers mostly terminate in the granule cells between the dorsal division and ventral division of the CN [3], [4], [20]. How are anteroventral CN neurons modulated by cortical activation? Although there is no answer to this question yet, some reports on corticofugal modulation of the auditory midbrain might give us some inspiration or hint. Anatomical studies suggest that majority of corticofugal fibers terminate in the external cortex of the inferior colliculus of the midbrain but only sparse but tonotopically-organizaed fibers terminate in the central nucleus [53]–[55]. Probably owing to these sparse fibers, focal cortical activation of the auditory cortex induces remarkable and frequency-specific BF shifts of central nucleus neurons [11], [15], [32]. Fifth, the auditory cortex also augments the microphonic potential of the inner ear in a frequency-specific manner [30], [31]. This suggests that the response of corresponding auditory nerve fibers increases. As the direct target of auditory nerve fiber, the changes in the auditory responses of CN neurons should reflect the changes in the cochlear microphonic potential. It would be important to clarify how much of the change in the auditory responses of CN neurons is attributable to change in cochlear microphonic potential following cortical activation. Last, CN neurons can be sorted into at least four classes according to their temporal response patterns or frequency tuning curves [56], [57]. One may argue that the impact of cortical activation on different classes of CN neurons is different. We speculate that corticofugal modulation of CN neurons should be independent of neuronal classes at least in frequency domain (i.e., frequency-specific remodelling such as BF shift) since the tonotopic organization of the CN is primarily determined by the innervations of the auditory nerves instead of the neuronal classes [58], [59].

## Materials and Methods

Sixty-six female C57 mice (Charles River Laboratories, Senneville, Canada) aged 4–6 weeks and weighing 12.6–19.3 g were used in this study. The methodologies involved in animal preparation, acoustic stimulation, electrical stimulation of the primary auditory cortex, action potential recordings in the CN and data processing are described elsewhere [15], [18], [60]–[62]. Essential portions of our methods are provided below. All protocols and procedures were approved by the Animal Care Committee of the University of Calgary (protocol number: M04044). The physiological experiments were performed in a soundproof chamber.

### Animal Preparation

Animals were anesthetised with a mixture of ketamine (85 mg/kg, ip.) and xylazine (15 mg/kg, ip.). Anaesthetic levels were regularly monitored by pinching the mouse tail with forceps. Additional doses of ketamine (17 mg/kg, ip.) and xylazine (3 mg/kg, ip.) were administered to maintain the anaesthetic level when necessary. Under anaesthesia, the mouse's head was immobilized with a custom-made head holder by rigidly clamping the palate and nasal/frontal bones. The mouth bar was adjusted to align the bregma and lambda points of the skull on the same plane. An incision was made along the midline of the scalp and subcutaneous tissue and muscle were removed to expose the skull. Holes measuring 2 mm in diameter were made in the skull with a dental drill to expose the right cerebellum superior to the CN (5.1–5.6 mm posterior to bregma, 2.3–2.8 mm lateral to the midline) and the right auditory cortex. Throughout surgery and subsequent physiological experiments, the mouse's body temperature was maintained at 37°C via a feedback-controlled heating pad.

### Acoustic Stimulation

Sixty-ms-long tone bursts with 5-ms rise/fall times were used as the acoustic stimuli. Pulses of sinusoidal waves were digitally synthesized, then converted into analog signals by an Enhanced Real-time Processor (RP2, Tucker-Davis Tech., Gainesville, FL, USA). The signals were fed to a digital attenuator (PA5) and speaker driver (ED1). The driver was designed to activate two separate electrostatic speakers. One speaker was placed 20 cm from and 45° to the right of the mouse's right ear to elicit the auditory responses from the right CN. In order that both ears receive similar tone stimulation, another speaker was identically positioned at the left ear to evoke auditory responses from the right AI. The output amplitudes of speakers were measured and calibrated at the positions of animal's right or left ear using a condenser microphone (Model 2520, Larson-Davis Laboratories, USA) and a microphone preamplifier (Model 2200C, Larson-Davis Laboratories). During calibration, the speaker was driven by 20 volt peak-to-peak sinusoidal bursts without attenuation. The amplitude of the tone bursts was expressed in decibel sound pressure levels (dB SPL, ref. 20 µPa). Frequencies and amplitudes of tone bursts were varied either manually or automatically using BrainWare software (Tucker-Davis Tech., Gainesville, FL, USA).

### Recording and Electrical Stimulation in the Primary Auditory Cortex (AI)

A tungsten electrode with tip impedance of ∼2 MΩ was perpendicularly inserted into the right auditory cortex. The electrode was initially connected to the recording system. Signals detected by the electrode were magnified 10,000 times after passing through a 16-channel pre-amplifier and then filtered by a bandpass of 0.3–10 kHz with a RA16 module (Tucker–Davis Tech., Gainesville, FL, USA). During the electrode penetration, a pure tone burst was continuously delivered at a rate of 1/s; frequency and amplitude of the tone burst were manually altered. Tone-evoked action potentials were commonly observed at approximately 300 µm below the brain surface (layers III-IV). The neuronal BF was immediately measured through manual alteration of the tone frequency and amplitude. On average, 5–8 penetrations were required to determine the location of the AI based on tonotopic organization of the auditory cortex [15], [16], [18]. The electrode was directed into the AI. The BF and minimum threshold of the recorded AI neurons were first measured through manual alteration of tone frequency and amplitude. Responses of AI neurons to a series of tone bursts (10 dB above the minimum threshold, 250 ms interval) were measured and stored using BrainWare software. This data allowed for calculation of the best frequency (BF) of the stimulated AI neurons at a later time. The electrode was disconnected from the recording system and connected to an A360 constant current isolator (World Precision Instruments, USA) that served as the source of electrical stimulation. The electrode was further inserted into the deep layers of the auditory cortex to an approximate depth of 700–800 µm below the brain surface (deep layers of the auditory cortex). An indifference electrode was placed on the cortex surface next to the stimulating electrode. Electrical pulses (monophasic, 0.1-ms, -500 nA constant current), generated by a Grass S88 stimulator (Astro-Medical, Inc., West Warwick, RI) and a A360 constant-current unit (World Precision Instrument, USA) were delivered to AI neurons through this electrode.

### Recording in the Anteroventral CN

Using a manipulator, a tungsten electrode of ∼2 MΩ impedance was dorsoventrally positioned in the right CN. The electrode was connected to the TDT 16-channel preamplifier. Pure tone bursts with manually altered frequency and amplitude were continuously delivered at a rate of 1/s as the electrode penetrated the cerebellum. Once tone-evoked action potentials were elicited, typically at a depth of 2.5–3.6 mm, the electrode was withdrawn and another penetration was made approximately 100 µm anterior to the previous entry point. The procedure was repeated until actions potentials were no longer evoked by tone stimulation. Further insertions, lateral or medial and then anterior, were made at 100 µm until the rostral boundary of the CN was established. Electrode penetration in a range of 25–300 µm caudal to the rostral boundary of the CN was required to measure the auditory responses of neurons in the anteroventral division. Once tone-evoked action potentials from the CN neurons were observed, the BF and minimum thresholds were audiovisually measured. The BF was defined as the frequency in which neurons exhibited their lowest response threshold. The minimum threshold was sound amplitude that just elicited CN neuronal responses to tone stimuli. Responses of CN neurons to a series of tone bursts (10 dB above the minimum thresholds) were measured and stored at a sampling frequency of 25 kHz. Tone frequency varied from 3 kHz to 40 kHz with a 1 kHz increment. An identical set of tone stimuli was repeated 15 times.

### Experimental Protocols

Once the BF and minimum thresholds of AI and CN neurons were measured, two experimental protocols were designed to examine the impact of the AI on the activities of CN neurons.

The first protocol examined time-related changes in the CN auditory responses following focal electrical stimulation of the AI (ES_AI_). A train of tone bursts with 500 ms interval was delivered to elicit frequency-dependent responses from the CN neurons. The train consisted of 11 tone bursts with frequencies varying from 5 kHz below to 5 kHz above the BFs (in 1 kHz increments) of the recorded CN neurons. To isolate the corticofugal effects, the tone amplitudes were set at the minimum thresholds of the CN neurons, thereby ensuring that neuronal responses were limited to 1–3 frequencies including neuronal BF. A train of tone bursts was delivered every 10 seconds. The responses of CN neurons to the first 50 trials (trains) served as control. Beginning with the 51^st^ trial, a single electrical pulse was delivered to the AI 500 ms prior to the onset of the first tone burst in a train. After 250 trials, ES_AI_ ceased despite the ongoing delivery of the trains. This procedure was allowed to continue until ES_AI_-evoked changes in the auditory responses of CN neurons mostly disappeared.

The second protocol examined frequency-dependent changes in the frequency tunings of CN neurons evoked by ES_AI_. The frequency tunings of CN neurons with a series of tone bursts 250 ms apart were sampled. These tones bursts had a frequency range of 3 kHz to 40 kHz that increased by 1 kHz increments and their amplitude was 10 dB above the minimum thresholds of the CN neurons. Each tone stimulus was repeated 15 times. Once the control was established, an electrical pulse was delivered to the AI at a rate of 4/s for 7 min. The frequency tuning of CN neurons was continuously sampled with the same tone set immediately following ES_AI_, then every 30 minutes until the ES_AI_-evoked changes in frequency tuning mostly disappeared.

In addition, two control groups were performed to examine the effects of sham AI stimulation and the effects of electrical stimulation of non-AI cortical area. In the group of sham stimulation, the stimulating electrode was placed in the AI but no electrical current was delivered during the observation period. In the group of electrical stimulation of non-AI cortical area, the stimulating electrode was placed in cortical area dorsal to the AI and the electrical stimulation was identical to that in protocol 1.

### Data Processing

Single-unit action potentials were isolated from the multi-unit recording system utilizing eight parameters of its waveform. These parameters included the peak, valley, spike height, spike width, peak time, valley time and two user-defined voltage values. Data collection was limited to neurons with stable spike waveforms over the entire recording session. Single-unit responses to a train of tone bursts were later displayed on dot rasters and/or peristimulus time histograms with a bin width of 1 ms.

In protocol one, the response latencies and spike numbers were calculated using data within a one-minute time frame (6 tone stimuli). The response latency of CN neurons was taken from an average of all spike latencies in one minute intervals. The latency of each spike was the time from the onset of tone to the spike. The spike number of the response was the count of all spikes within the same time frame.

In protocol two, the excitatory frequency-response curve was derived from the response magnitudes of a single neuron to various tone frequencies. The BF was the frequency to which a given neuron showed the largest response magnitude.

In two control groups, data were sample with protocol 1. The spike numbers and averaging response latencies were calculated using data within a 10-minute window.

### Histology

Once the physiological portion of the experiment was completed, a 1 mA electrical current was applied through the recording electrode to the recording site in the CN for 20 s. The animal was administered a cardiac perfusion of 10 ml of physiological saline followed by a mixture of 4% paraformaldehyde in a 0.1 M phosphate buffer with a pH of 7.4. The brain was then removed from the cranium, fixed by immersion in 4% paraformaldehyde with the same composition as perfusion solution and stored in PBS containing 20% sucrose at 4°C. After embedding in O.C.T (optimal cutting temperature) compound and frozen in 2-methylbutane, 40 µm coronal brainstem sections were made with a cryostat. The tissue sections were mounted onto glass slides and stained via the Nissl technique. The electrolytic lesion was examined under a light microscope to verify the recording electrode position.

### Statistics Analysis

Data were expressed as mean ± standard deviation. Data between matched and unmatched neurons sampled with protocol 1 were compared with a *t-test*. A *t-test* was also employed to examine the difference in the spike numbers and response latencies before and after the electrical stimulation, before and after injection of anaesthetic drugs. A *p* value of less than 0.05 was considered to be statistically significant.
